# Predicting non-improvement of symptoms in daily mental healthcare practice using routinely collected patient-level data: a machine learning approach

**DOI:** 10.3389/fpsyt.2023.1236551

**Published:** 2023-09-25

**Authors:** Katinka Franken, Peter ten Klooster, Ernst Bohlmeijer, Gerben Westerhof, Jannis Kraiss

**Affiliations:** Department of Psychology, Health and Technology, Faculty of Behavioural, Management and Social Sciences, University of Twente, Enschede, Netherlands

**Keywords:** machine learning, mental disorder, well-being, psychopathology, prediction, non-improvement

## Abstract

**Objectives:**

Anxiety and mood disorders greatly affect the quality of life for individuals worldwide. A substantial proportion of patients do not sufficiently improve during evidence-based treatments in mental healthcare. It remains challenging to predict which patients will or will not benefit. Moreover, the limited research available on predictors of treatment outcomes comes from efficacy RCTs with strict selection criteria which may limit generalizability to a real-world context. The current study evaluates the performance of different machine learning (ML) models in predicting non-improvement in an observational sample of patients treated in routine specialized mental healthcare.

**Methods:**

In the current longitudinal exploratory prediction study diagnosis-related, sociodemographic, clinical and routinely collected patient-reported quantitative outcome measures were acquired during treatment as usual of 755 patients with a primary anxiety, depressive, obsessive compulsive or trauma-related disorder in a specialized outpatient mental healthcare center. ML algorithms were trained to predict non-response (< 0.5 standard deviation improvement) in symptomatic distress 6 months after baseline. Different models were trained, including models with and without early change scores in psychopathology and well-being and models with a trimmed set of predictor variables. Performance of trained models was evaluated in a hold-out sample (30%) as a proxy for unseen data.

**Results:**

ML models without early change scores performed poorly in predicting six-month non-response in the hold-out sample with Area Under the Curves (AUCs) < 0.63. Including early change scores slightly improved the models’ performance (AUC range: 0.68–0.73). Computationally-intensive ML models did not significantly outperform logistic regression (AUC: 0.69). Reduced prediction models performed similar to the full prediction models in both the models without (AUC: 0.58–0.62 vs. 0.58–0.63) and models with early change scores (AUC: 0.69–0.73 vs. 0.68–0.71). Across different ML algorithms, early change scores in psychopathology and well-being consistently emerged as important predictors for non-improvement.

**Conclusion:**

Accurately predicting treatment outcomes in a mental healthcare context remains challenging. While advanced ML algorithms offer flexibility, they showed limited additional value compared to traditional logistic regression in this study. The current study confirmed the importance of taking early change scores in both psychopathology and well-being into account for predicting longer-term outcomes in symptomatic distress.

## 1. Introduction

### 1.1. Prevalence and impact of psychiatric disorders

Worldwide, around one in eight people has one or more mental disorders (1). Mental disorders are the leading cause of years lived with disability (YLDs), accounting for one in every six YLDs globally (1). They contribute significantly to a lack of quality of life (2) and the direct and indirect economic and societal costs are substantial (1). Depression and anxiety alone result in the loss of nearly US$ 1 trillion and 12 billion working days every year (3). The increasing demand for care in combination with limited treatment effects puts pressure on waiting lists in mental healthcare (4). Insights in predicting who is less likely to improve early in treatment would be helpful to make treatment more efficient, reduce waste of financial and human resources and tailor treatment to the individual (5–7).

### 1.2. Predicting treatment effects

Studies show that 60% of patients with a mental disorder do not benefit from evidence-based treatments (8–12). At present, no convincing evidence has been found for a difference in treatment effect for any specific treatment, neither for mood disorders (13, 14) nor anxiety disorders (14, 15). Norcross and Lambert (16) argue that fitting psychotherapy to patient characteristics is necessary for treatment success. Clinical practice, however, shows that a DSM-classification alone does not give sufficient direction to appropriate treatment (17–19). This underlines the relevance of adopting a more transdiagnostic approach in clinical practice and searching for predictors across the main diagnoses. Early identification of non-responders can increase treatment effectiveness as it may support personalized treatment recommendations (20).

Mental disorders are complex and trajectories of treatment can depend on many factors, making the prediction of treatment outcomes challenging. Previous studies have incidentally found several predictors for treatment outcomes in various populations, including sociodemographic features (age, gender, employment status), symptom severity, emotion regulation abilities, problem duration, level of functioning, interpersonal problems, prior treatments, comorbidity of personality disorders or medical conditions, treatment non-adherence and alliance [e.g., (6, 7, 21–33)]. However, no consistent pre-treatment characteristics have been identified that reliably predict treatment outcomes (34, 35).

### 1.3. Importance of analyzing longitudinal data from the real-world psychiatric context

Findings about predictors for treatment outcomes often stem from data from randomized controlled trials (RCTs), which may be problematic for several reasons. First, only a selective and limited number of potential predictors are usually included in randomized controlled trials (RCTs), only allowing for limited conclusions about what predictors are relevant for treatment outcomes. Second, RCTs often do not meet the required sample size needed for detecting significant predictors (36–41). Third, many RCTs, especially efficacy trials, tend to have rather strict in- and exclusion criteria and controlled study procedures. While the use of such criteria leads to relatively high internal validity, it may decrease external validity and limit generalizability to patient populations treated in daily clinical practice (42–44).

Considering that most patients are not treated in RCTs, but in naturalistic clinical institutions, using real-world clinical data to identify predictors of treatment outcomes is likely to be more externally valid (45). Large observational studies using data collected in the real-world context may be a valuable alternative to develop more generalizable prediction models (28). Longitudinal routinely collected patient-reported outcome data of psychopathology and well-being are increasingly available that provide information about treatment outcomes [e.g., (46, 47)]. For instance, electronic health records (EHRs) of psychiatric patients contain large amounts of potentially useful clinical information. However, despite increased external validity, such routinely collected data presents challenges as well. Predictive features are heterogeneous and may interact with each other in ways that traditional statistical models may not be able to capture. By including a larger number of features there is also a risk for overfitting. In addition, using real-world data is often challenging, especially due to high attrition and missing data rates.

### 1.4. The potential of machine learning

Recent improvements in computational power and the refinement of the applications of machine learning (ML) technologies have been suggested to offer possibilities to develop robust and generalizable prediction models for treatment response using real-world data (18, 48, 49). ML has shown promise within clinical psychology in helping to understand large-scale health data (50–55). ML is a subfield of artificial intelligence that involves the development of algorithms and statistical models that enable computers to learn and make predictions or decisions based on data without being explicitly programmed to do so (56).

ML can predict treatment effects using high-quality data such as patient characteristics and questionnaire scores over time [e.g., (55, 57, 58)]. The techniques used in building ML models depend on the type of data and can be based on supervised learning, unsupervised learning, and reinforcement learning. Supervised learning, as applied in current study, involves training a model on labeled data, where the desired output is already known. The ultimate goal is to build a model that can accurately predict future outcomes (59). Aafjes-van Doorn, Kamsteeg, Bate, and Aafjes (60) systematically reviewed 51 studies of ML in psychotherapy and concluded that most model development studies used supervised learning techniques to classify or predict labeled treatment process or outcome data, whereas some used unsupervised techniques to identify clusters in the unlabeled patient or treatment data.

In ML models, the main statistic of interest is the prediction accuracy of the algorithm in a hold-out sample. The hold-out sample is a random subset of the original dataset that is held back and not used during training. For categorical outcomes the accuracy is usually reported as the accuracy, sensitivity (or recall) and specificity, and area under the curve (AUC) computed from the confusion matrix of the predicted against the observed labels of the observations.

Application of ML has various potential advantages above traditional statistical methods. First, by employing robust statistical and probabilistic techniques, ML has the ability to make predictions regarding treatment effects, enabling the comprehension of complex, integrated datasets consisting of heterogeneous features (57, 60). Second, ML methods require less restrictive assumptions regarding the non-linear relationship of high-dimensional data and the skewed distribution of features (61). The potential of ML has been demonstrated by improved accuracy compared to regular methods such as regression (62, 63). Third, the application of cross-validation techniques, which are common in ML methods but usually not applied in traditional prediction analyzes such as significance-based regression, reduces the risk for overfitting (64). Fourth, ML increases the generalizability of the predictions since some ML algorithms might perform better than traditional analysis techniques in complex datasets involving many features (65).

### 1.5. Predicting non-improvement by ML using outcome data of psychopathology and well-being

Real-world mental health data have been used in various ML applications, such as modeling disease progression (66), predicting disease deterioration (67), predicting risk factors for adverse outcomes, such as mortality, readmission or prolonged length of stay (68) as well as predicting treatment outcomes (69). However, research predicting outcomes using real-world clinical data is still scarce. Some studies have shown that compared to traditional research methods ML can increase prediction accuracy using sociodemographic, clinical and biological data (19, 63, 64, 70–74). However, ML has not often been applied to the routine collection of patient-level outcome data in combination with sociodemographic and clinical data.

Hence, the objective of the current study is to evaluate and compare the performance of different ML models in predicting treatment outcomes in an observational sample of patients treated in routine specialized mental healthcare. This will be done by predicting non-improvement in psychopathology 6 months after start of treatment in a group of patients with anxiety and mood disorders. A range of routinely available clinical, demographic and self-reported outcome features will be used to predict treatment outcomes. Several models are explored, such as those involving the incorporation of change scores early in treatment as supplementary predictors, and models that are trained on a reduced set of features using feature reduction techniques.

## 2. Methods

### 2.1. Study design and data collection

The present study concerned an exploratory machine learning based prediction analysis of routinely collected observational longitudinal quantitative data. The recommendations for reporting machine learning analyzes in clinical research (75) were followed. We used data collected in the context of routinely collected patient-level outcome data of psychopathology and well-being, a standardized service to measure treatment effects. Patients in a mental healthcare center in the Netherlands completed online questionnaires every 3 months from the initial interview to end of treatment. Data were collected before start of treatment (T0), and three (T1), six (T2), nine (T3), and 12 (T4) months after treatment commenced. Invitations to complete the questionnaires were sent automatically and data from the completed questionnaires were stored anonymously by an independent data controller in a database generated for this longitudinal study. The data were gathered between March 2015 and November 2019. About 19% (*n* = 145) were lost to 3-month follow-up, 34% (*n* = 254) did not complete the six-month follow-up assessment, and about 58% (*n* = 439) did not complete the 12-month follow-up.

Patients provided passive informed consent for their anonymized data to be used for scientific research. As data were collected in the context of regular care and only anonymized data were analyzed, the study did not require medical ethical approval according to Dutch law. Inclusion criteria were: (1) aged between 18 to 65 years, (2) full completion of the questionnaires on the same day, and (3) diagnosed by depressive, bipolar, anxiety, trauma related or obsessive-compulsive disorder. The diagnosis was based on an extensive interview by a licensed clinical psychologist or psychiatrist. The diagnosis and related (evidence- and practice-based) treatment options were discussed and confirmed in a multidisciplinary team.

### 2.2. Baseline features

An overview of all available baseline features that were included in the models can be found in Table 1. These include sociodemographic (e.g., gender, age), diagnostic (e.g., main diagnosis, comorbidity), and clinical characteristics of patients (e.g., number of treatments in the past, social problems). One additional clinical feature was created that was labeled as treatment intensity. This feature represents the ratio of number of treatments in the past and total duration of past treatments. Routinely collected self-reported psychological features included the total and subscales scores of the Outcome Questionnaire [OQ-45; (76)] and the Mental Health Continuum-Short Form [MHC-SF; (77, 78)]. The OQ-45 is a 45-item self-report measure of psychopathology and includes four subscales, namely symptomatic distress (e.g., “I’m anxious”), interpersonal relations (e.g., “Often I have fights”), somatic complaints (e.g., “I tire quickly”), and social roles performance (e.g., “I feel like I’m not doing well with my work”). Items are answered on a five-point Likert scale ranging from 0 (*never*) to 4 (*almost always*). Previous studies have shown that the OQ-45 is a reliable and valid instrument across different cultural contexts (76, 79, 80). The 14-item MHC-SF measures the presence of different well-being dimensions during the past month on three subscales: emotional (e.g., “Feeling satisfied with life”), social (e.g., “Feeling that you belong to a community”), and psychological well-being (e.g., “Feeling that your life as a sense of direction or meaning to it”). Items are answered on a six-point Likert scale ranging from 0 (*never*) to 5 (*every day*). The MHC-SF has shown good psychometric properties in the general population [e.g., (77, 78)] and in clinical groups (81). In total, 41 baseline features were included in the models.

**Table 1:** Overview of baseline features.

Sociodemographic	Psychological
Age Gender (male/female) Education (low/moderate/high) Marital status (no partner/partner/other)	OQ-45 Total score OQ-45 Symptomatic distress OQ-45 Anxiety and somatic distress OQ-45 Interpersonal relationships OQ-45 Social role adjustment MHC-SF Total score MHC-SF Emotional well-being MHC-SF Social well-being MHC-SF Psychological well-being GAF score
Diagnostic	Clinical
Main diagnosis (depressive disorder, anxiety disorder, bipolar disorder, OCD, traumatic disorder) First comorbidity (no/yes) Second comorbidity (no/yes) Somatic comorbidity (no/yes)	Axis II problem (no/yes) Axis IV financial problem (no/yes) Axis IV relationship problems (no/yes) Axis IV social problems (no/yes) Axis IV work problems (no/yes) Number of treatments in the past (0–4/5–10/10+) Years since first time enrolled (0–3/3–10/10+) Sum of previous enrollments in years (0–2/2–5/5+) Log-transformed treatment intensity^a^

^a^Treatment intensity was calculated as the ratio of number of treatments in the past and total duration of past treatments.

### 2.3. Response variable

Non-improvement on the OQ-45 total scores at six-month follow-up was used as binary response variable. Cases were labeled as ‘not improved’ if the change from baseline in the symptomatic distress scale of the OQ-45 6 months after baseline was smaller than half a standard deviation (0.5 SD). The choice of this cut-off is motivated by a previous systematic review of 38 studies, suggesting that half a standard deviation consistently reflected a minimally important difference for health-related quality of life instruments across studies (82). Half a standard deviation also corresponds with a medium effect size according to Cohen’s conventional rule of thumb (83). The reason to use improvement at six-month follow-up as response variable, was that missing data become too high at later follow-up points and because 6 months was considered a time period long enough to be clinically relevant. Besides, hardly any additional average treatment effects were observed after that time in the dataset.

### 2.4. Preprocessing

Descriptive analyzes were done in the statistical package for social sciences (SPSS) version 27 (84). All other ML analyzes were conducted in R (85) using the caret R-package (86). Data, syntax and output files can be found on the Open Science Framework website (https:osf.io/xwme4/).

All categorical features were dummy coded and continuous features were visually checked for approximate normal distribution. The feature ‘treatment intensity’ was log-transformed, since it was not normally distributed and right-skewed. Cases that did not complete the OQ-45 at 6 months after baseline were removed. Only complete cases were used, since imputing the response variable might overestimate the performance of the ML algorithms, as common imputation techniques (e.g., random forest) would be similar to what ML algorithms would use to predict non-improvement at follow-up. After data preprocessing and cleaning, the remaining data was randomly split into a training (70%) and hold-out sample (30%). Next, missing baseline data (0.8%) was globally imputed (before conducting k-fold cross-validations) and separately for training and hold-out data, using random forest imputation (87).

### 2.5. Machine learning models and model performance

The goal of ML is to identify patterns in observed high complex data in high dimensional settings, make accurate predictions or classifications, and improve their performance over time by learning from new data [e.g., (57, 58, 63, 88–90)]. ML algorithms involve three main components, which are (1) a model, (2) data for training, testing and validation, and (3) an optimization algorithm. The model represents the data and relationships between features. The training data is used to optimize model weights using cross-validation (CV) to minimize error or loss, while the optimization algorithm finds the optimal values of the model weights. ML algorithms are conducted in two steps: training and testing. During training, the objective is to find a balance between identifying specific patterns in the patient data and preventing overfitting (training data so well that it negatively affects its performance on new data, which occurs when the algorithm fits too closely to the random noise in the data). In the test phase, the accuracy of the predictions made by the algorithm is computed by comparing the predictions made for new data with the actual values observed in the new sample. CV optimizes the ML model by assessing skills of the ML model and testing its performance (or accuracy) in new data later.

Different ML algorithms were compared to predict non-improvement at six-month follow-up. The following algorithms were used: Logistic regression (LR), random forest (RF), support vector machine (SVM) with linear, radial and polynomial kernels, and gradient boosting machine (GBM). These algorithms differ in their underlying principles and modeling techniques. LR focuses on estimating probabilities based on linear relationships, RF combines decision trees for predictions, SVM find optimal hyperplanes for classification, and GBM sequentially build models to minimize prediction error. The rationale for choosing these algorithms was to be able to compare this study with previous studies that used similar algorithms [e.g., (19, 74)]. Furthermore, we not only wanted to include flexible and less interpretable algorithms (e.g., GBM or SVM), but also techniques that are easier to interpret, while being less flexible (91).

All models were trained on the training set using repeated k-fold cross-validation with 10 folds and 10 repetitions (90). As the response variable was imbalanced, up-sampling was used for training purposes, which randomly replicates instances of the minority class. We explored the effect of class imbalance before applying up-sampling. If no up-sampling was used models performed comparably well in terms of overall accuracy, but were not useful because the sensitivity was extremely high (often higher than 90%), while the specificity was often extremely low (often about 10–20%). We therefore decided to use up-sampling techniques for training the model, in order to create models that are more balanced in terms of sensitivity and specificity.

Using class weights (i.e., imposing a heavier cost for errors made in the minority class) was tested as an alternative to up-sampling, but did not lead to a substantially different performance.

Depending on the model, different hyperparameters were tuned for training the models. For RF models, the number of features used at each split was tuned. For linear SVM, the C hyperparameter was tuned, for SVM with radial basis function kernel the C and sigma parameters were tuned, for SVM with polynomial basis function the C, degree, and scale parameters were tuned, and for GBM number of iterations and complexity of the tree were tuned, while shrinkage and minimum number of training set samples in a node to commence splitting was held constant at 0.1 and 10, respectively. Model training was done in different settings. First, models were fitted that only included baseline features (T0). Second, models were fitted that additionally included three-month change scores in OQ-45 (psychopathology) and MHC-SF (well-being) subscales and total scores. Change scores were included in the second setting, because early improvements in treatment have been shown to be a strong and unique indicator for ongoing improvement at a later moment across a range of psychiatric disorders (92–94). If such a model would perform substantially better, it would be of added value for practice to (additionally) use this model some months after the treatment started to make more accurate predictions.

Third, additional feature reduction was used in both settings, because this might avoid overfitting and lead to better generalizability and increased performance on the test set. The practical usefulness of a model would increase if a reduced set of features yields comparable or even superior performance in predicting non-improvement. Least absolute shrinkage and selection operator regression (LASSO) was used to reduce the number of features. LASSO has the advantage of shrinking less relevant weights to zero, allowing to use it to reduce the number of features (90, 95, 96). In total, this resulted in four settings used for training the models: (1) no change scores and not reduced, (2) no change scores and reduced, (3) change scores and not reduced, and (4) change scores and reduced.

The trained models were then validated in the hold-out sample using a default probability cut-off of 0.5 (82). This means that every case that had a probability higher than 50% of not being improved, was classified as ‘not improved’. Performance of all models was evaluated using balanced accuracy, sensitivity, specificity, and area under the curve (AUC). Sensitivity, also known as True Positive Rate (TPR) or recall, focuses on the model’s ability to correctly detect positive instances whereas specificity, also known as True Negative Rate (TNR), assesses the model’s ability to correctly identify negative instances. Both sensitivity and specificity refer to a specific prediction threshold of the outcome. The AUC, on the other hand, provides a global evaluation, capturing the model’s performance across the entire range of threshold choices. AUC thus provides a holistic view of performance, independent of thresholds, making it a valuable measure to assess the overall discriminatory power of our binary classification model (improvement versus non-improvement). Therefore, the AUC was used as the primary evaluation measure in this study. Guidelines for interpreting AUC scores suggest that scores from 0.5 to 0.59 can be seen as extremely poor, from 0.60 to 0.69 as poor, 0.70 to 0.79 as fair, 0.80 to 0.89 as good and > 0.90 as excellent (97).

To be better able to interpret the models and for reasons of conciseness, we additionally determined the top five most important features in the hold-out sample of each model in the four different settings. Feature importance was determined using the varImp evaluation function from the caret package, a generic calculation method and analysis technique for statistical modeling. It evaluates the impact of each predictor feature by assessing how much the model’s performance deteriorates when a particular feature is removed. By measuring the relative contribution of the features, it helps in understanding the ranking of influence on the prediction of non-improvement, ensuring further model optimization. Depending on the type of model, different metrics are used to determine feature importance [for an overview, see Kuhn, (86)].

## 3. Results

### 3.1. Sample

At baseline, 755 patients receiving outpatient treatments within multidisciplinary teams consisting of psychologists, psychiatrists, nurses and art therapists, were included in the dataset. Most patients were female, followed lower (43%), intermediate (37.1%) or higher (19.9%) vocational education, and lived with a partner and children (see Table 2). Almost one third had social, relation and/or work problems. The respondents were classified into five common psychopathological groups based on their primary diagnosis: depressive disorder (*n* = 417; 55.2%), bipolar disorder (*n* = 79; 10.5%), anxiety disorder (*n* = 114; 15.1%), trauma related disorder (*n* = 115; 15.2%) or obsessive-compulsive disorder (*n* = 30; 4.0%). Most patients had comorbid disorders ranging from attention deficit hyperactivity disorder (ADHD), depression, anxiety, trauma or addiction, and/or had personality problems respectively: depressive disorder (5.0%; 31.3%), bipolar disorder (6.3%; 1.3%), anxiety disorder (8.8%; 29.8%), trauma related disorder (14.8%; 32.2%) or obsessive-compulsive disorder (OCD; 10.0%; 26.7%).

**Table 2:** Major characteristics of respondents (*N* = 755).

	Depression (*n* = 417) (55.2%)		Bipolar (*n* = 79) (10.5%)		Anxiety (*n* = 114) (15.1%)		Trauma (*n =* 115) (15.2%)		OCD (*n =* 30) (4.0%)		Total (*N =* 755)	
Gender *n* (%)												
Male	190	(45.6)	32	(40.5)	45	(39.5)	35	(30.4)	8	(26.7)	310	(41.1)
Female	227	(54.4)	47	(59.5)	69	(60.5)	80	(69.6)	22	(73.3)	445	(58.9)
Age												
Mean	46.0		45.6		39.3		41.0		36.8		43.8	
Range	20–65		25–64		21–62		19–63		21–65		19–65	
SD	10.8		10.0		10.4		10.6		12.0		11.1	
Level of education *n* (%)^a^												
Low	182	(46.8)	13	(19.1)	43	(39.4)	60	(53.6)	6	(20.7)	304	(43.0)
Moderate	143	(36.8)	29	(42.6)	45	(41.3)	33	(29.5)	12	(41.4)	262	(37.1)
High	64	(16.5)	26	(38.2)	21	(19.3)	19	(17.0)	11	(37.9)	141	(19.9)
Marital status *n* (%)												
Single without children	77	(18.9)	14	(19.7)	17	(14.9)	30	(26.5)	2	(6.7)	140	(19.0)
Single with children	30	(7.4)	6	(8.5)	13	(11.4)	16	(14.2)	1	(3.3)	66	(9.0)
Married without children	93	(22.9)	12	(15.2)	22	(19.3)	17	(15.0)	9	(30.0)	153	(20.8)
Married with children	161	(39.6)	33	(46.5)	36	(31.6)	33	(29.2)	11	(36.7)	274	(37.3)
Other	46	(11.3)	6	(8.5)	26	(22.8)	17	(15.0)	7	(23.3)	102	(13.9)
Comorbid society problems *n* (%)												
House problem	18	(4.3)	0	(0)	2	(1.8)	2	(1.7)	2	(6.7)	24	(3.2)
Work problem	112	(27.0)	14	(17.7)	31	(27.4)	29	(25.2)	6	(20.0)	192	(25.3)
Relation problem	109	(26.3)	3	(3.8)	21	(18.6)	28	(24.3)	3	(10.0)	164	(21.8)
Social problem	126	(30.4)	10	(12.7)	30	(26.5)	33	(28.7)	6	(20.0)	205	(27.3)
Financial problem	59	(14.2)	1	(1.3)	11	(9.7)	13	(11.3)	3	(10.0)	87	(11.6)
Somatic problem	61	(14.6)	3	(3.8)	21	(18.4)	8	(7.0)	2	(6.7)	95	(12.6)
Comorbid diagnosis *n* (%)												
None	273	(65.5)	58	(73.4)	67	(58.8)	42	(36.5)	19	(63.3)	459	(60.8)
Two or more	21	(5.0)	5	(6.3)	10	(8.8)	17	(14.8)	3	(10.0)	56	(7.4)
Personality problems	130	(31.3)	1	(1.3)	34	(29.8)	37	(32.2)	8	(26.7)	225	(29.8)
Nature all comorbid diagnoses *n* (%)												
ADHD	24	(5.8)	12	(15.2)	6	(5.3)	16	(13.9)	1	(3.3)	59	(7.8)
Depression	–	–	–	–	25	(21.9)	27	(23.5)	6	(20.0)	58	(7.7)
Anxiety	32	(7.7)	0	(0)	–	–	4	(3.5)	1	(3.3)	37	(4.9)
Trauma	34	(8.2)	5	(6.3)	6	(5.3)	–	–	0	(0)	45	(6.0)
Addiction	19	(4.6)	2	(2.5)	2	(1.8)	6	(5.2)	1	(3.3)	30	(4.0)
Other	35	(8.4)	2	(2.5)	8	(7.0)	20	(17.4)	2	(6.7)	67	(8.9)

^a^Low = primary school, lower vocational education; moderate = secondary school, intermediate vocational education; high = higher vocational education, university.

### 3.2. Psychopathology and well-being per diagnosis over time

**Figure 1:**
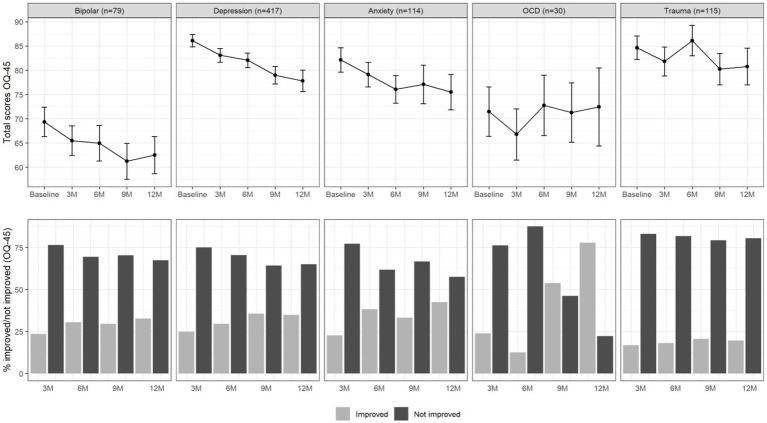
Total OQ-45 scores (upper panel) and percentage of improved and not improved patients (lower panel) per diagnosis group and over time. The error bars in the upper panel represent 95% confidence intervals.

For descriptive purposes, Figure 1 shows the average OQ-45 symptomatic distress scale scores over the 12-month time span for the different diagnostic categories, as well as the percentages of patients who did or did not improve by more than half an SD compared to baseline. For patients with depressive disorder, a continuous improvement from baseline to 12-month follow-up seemed to be present in the total OQ-45 scores. For patients with anxiety disorder, it seemed that on average no improvement was present after six-month follow-up. The binary improvement data suggests that the largest proportion of improvement happened within the first 3 months. The increase in percentage improved after this point seemed very small for all diagnostic groups. The percentage of improved patients in the trauma-related disorder group seemed especially small.

**Figure 2:**
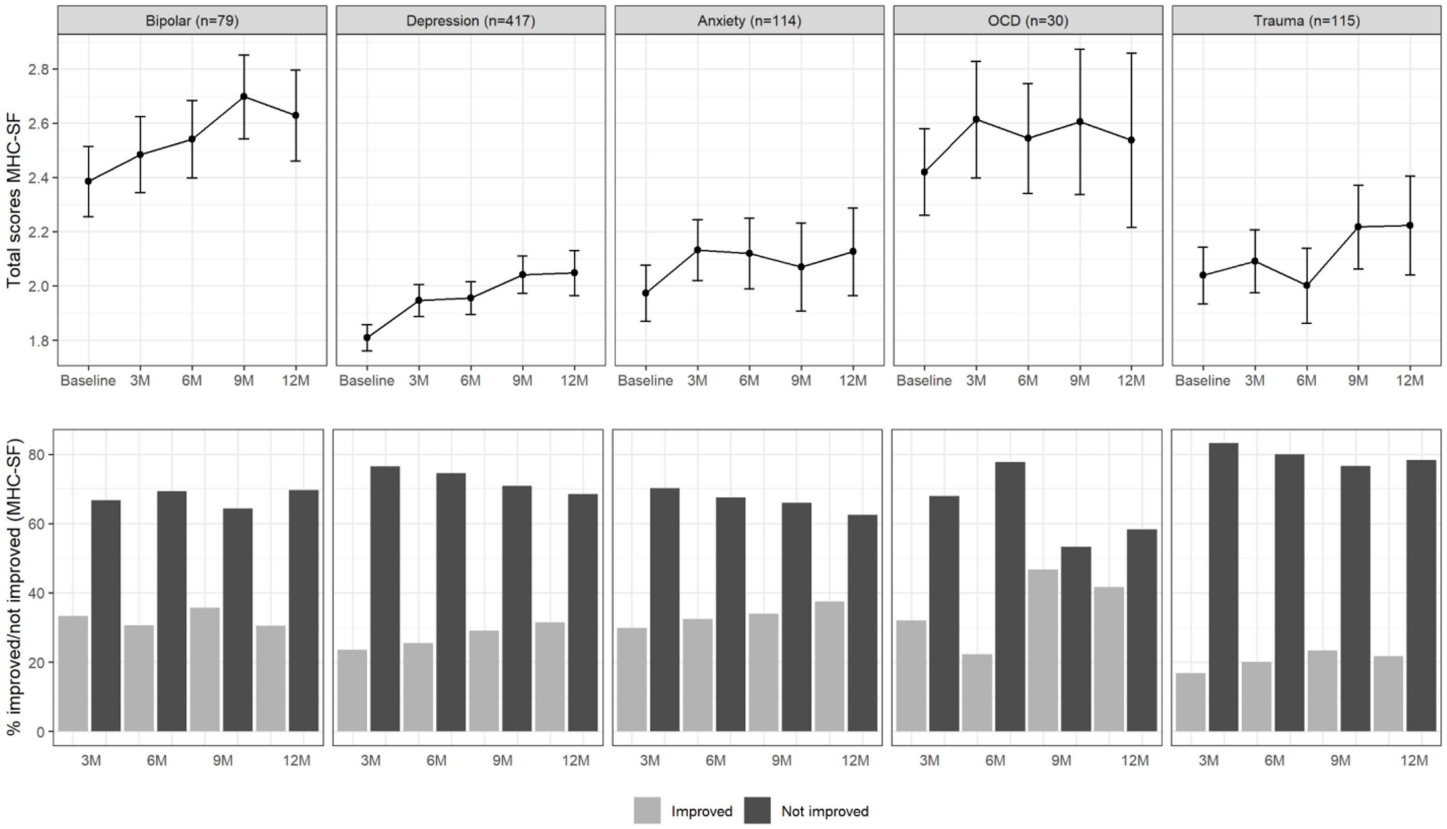
Total MHC-SF scores (upper panel) and percentage of improved and not improved patients (lower panel) per diagnosis group and over time. The error bars in the upper panel represent 95% confidence intervals.

Figure 2 summarizes the course of total well-being scores over the period of 12 months and the proportion of patients that improved (> 0.5 SD) in well-being. Overall, a similar picture emerged. Improvements in well-being appeared to happen mainly within the first 3 months, while the increase in improvements after this point remained rather small.

### 3.3. Feature reduction

Table 3 gives an overview of features that were included in the models without change scores and with change scores after LASSO regression was applied as additional preparatory step. In models without change scores, the only psychological feature of the 16 remaining features after feature reduction was the baseline total score of the OQ-45, while all others were demographic, diagnostic and clinical features. In models with change scores of the remained 13, psychological features of both the OQ-45 and the MHC-SF turned out to be of interest.

**Table 3:** Overview of features that were included after feature reduction was applied using LASSO regression.

Model without change scores (*k* = 16)	Model with change scores (*k* = 13)
OQ-45 symptomatic distress	OQ-45 symptomatic distress
Gender	Change score OQ-45 interpersonal relations
Working problems	Change score OQ-45 somatic complaints
Living problems	Change score OQ-45 symptomatic distress
Log-transformed treatment intensity^a^	Change score MHC-SF total score
Education: moderate	Change score MHC-SF emotional well-being
Living situation: no partner	Main diagnosis: trauma
Living situation: other	Main diagnosis: anxiety
Comorbidity	Second comorbidity
Second comorbidity	Living situation: other
Main diagnosis: trauma	Working problems
Main diagnosis: anxiety	Living problems
Sum of previous enrollments in years: 0–2	Social problems
Sum of previous enrollments in years: 5+	–
Number of treatments in the past: 1–4	–
Number of treatments in the past: 5–10	–

MHC-SF, Mental Health Continuum-Short Form; OQ-45, Outcome Questionnaire. All change scores refer to change from baseline to 3-month follow-up.

### 3.4. Predicting improvement at 6 months

In the training set, 70% of cases did not improve and in the hold-out sample 71% of cases did not improve. An overview of the performance of all models under the four different settings can be found in Table 4. Overall, the models performed best when change scores were included. In settings in which early change scores were included (from 0 to 3 months), the highest overall performance on the training set was obtained (AUC range: 0.79–0.84). The models in this setting also performed best on the hold-out sample (AUC range: 0.69–0.73). The best performing overall model in the hold-out sample in settings with change scores included was gradient boosting (AUC = 0.73). The models performed relatively poor in settings without change scores. In the training set, modest AUC values were found in these settings, ranging from 0.67 to 0.73. The best performance in the hold-out sample when no change scores were included was found for logistic regression (AUC = 0.63). Overall, these findings suggest that including change score substantially improves model performance in this dataset. An overview of all final hyperparameters after model training can be found in Table 5.

**Table 4:** Model performance metrics of the six algorithms under different conditions in the training and hold-out sample.

		Training sample (*n* = 344)				Hold-out sample (*n* = 146)			
Setting	Algorithm	ACC_Bal_	Sens	Spec	AUC	ACC_Bal_	Sens	Spec	AUC
No change scores, not reduced	Logistic regression	0.61	0.66	0.56	0.68	0.59	0.64	0.54	0.63
	Random forest	0.62	0.67	0.57	0.67	0.52	0.59	0.44	0.58
	SVM (linear)	0.63	0.66	0.60	0.68	0.58	0.65	0.51	0.60
	SVM (radial)	0.62	0.70	0.54	0.69	0.56	0.65	0.47	0.62
	SVM (polynomial)	0.62	0.69	0.56	0.69	0.54	0.59	0.49	0.58
	Gradient boosting	0.61	0.68	0.54	0.67	0.54	0.71	0.37	0.58
No change scores, reduced	Logistic regression	0.66	0.69	0.64	0.73	0.59	0.63	0.56	0.62
	Random forest	0.65	0.68	0.62	0.71	0.56	0.61	0.51	0.58
	SVM (linear)	0.66	0.67	0.65	0.73	0.58	0.58	0.58	0.61
	SVM (radial)	0.66	0.70	0.63	0.73	0.56	0.61	0.51	0.59
	SVM (polynomial)	0.67	0.67	0.67	0.73	0.61	0.60	0.63	0.60
	Gradient boosting	0.65	0.67	0.63	0.72	0.59	0.67	0.51	0.62
Change scores, not reduced	Logistic regression	0.70	0.76	0.64	0.79	0.65	0.77	0.53	0.69
	Random forest	0.68	0.88	0.47	0.80	0.65	0.93	0.37	0.71
	SVM (linear)	0.72	0.76	0.67	0.80	0.67	0.76	0.58	0.69
	SVM (radial)	0.72	0.77	0.67	0.80	0.63	0.70	0.56	0.71
	SVM (polynomial)	0.72	0.75	0.68	0.81	0.63	0.73	0.53	0.68
	Gradient boosting	0.73	0.77	0.69	0.81	0.66	0.77	0.56	0.71
Change scores, reduced	Logistic regression	0.74	0.78	0.70	0.83	0.65	0.74	0.56	0.69
	Random forest	0.74	0.81	0.66	0.83	0.64	0.74	0.54	0.69
	SVM (linear)	0.74	0.77	0.71	0.84	0.66	0.74	0.58	0.69
	SVM (radial)	0.73	0.76	0.69	0.81	0.63	0.72	0.54	0.70
	SVM (polynomial)	0.74	0.76	0.71	0.84	0.67	0.76	0.58	0.70
	Gradient boosting	0.74	0.78	0.71	0.83	0.64	0.77	0.52	0.73

**Table 5:** Final hyperparameters used for prediction in the hold-out sample after model training.

Setting	Algorithm	Hyperparameter
No change scores, not reduced	Logistic regression	NA
	Random forest	mtry = 1
	SVM (linear)	C = 0.01
	SVM (radial)	C = 0.5, sigma = 0.02
	SVM (polynomial)	C = 0.25, degree = 3, scale = 0.01
	Gradient boosting	nTrees = 150, ID = 1, shrinkage = 0.1, NT = 10
No change scores, reduced	Logistic regression	NA
	Random forest	mtry = 1
	SVM (linear)	C = 0.01
	SVM (radial)	C = 0.25, sigma = 0.04
	SVM (polynomial)	C = 0.25, degree = 2, scale = 0.01
	Gradient boosting	nTrees = 150, ID = 1, shrinkage = 0.1, NT = 10
Change scores, not reduced	Logistic regression	NA
	Random forest	mtry = 2
	SVM (linear)	C = 0.01
	SVM (radial)	C = 0.25, sigma = 0.01
	SVM (polynomial)	C = 0.25, degree = 1, scale = 0.01
	Gradient boosting	nTrees = 50, ID = 1, shrinkage = 0.1, NT = 10
Change scores, reduced	Logistic regression	NA
	Random forest	mtry = 1
	SVM (linear)	C = 0.01
	SVM (radial)	C = 0.5, sigma = 0.06
	SVM (polynomial)	C = 0.5, degree = 1, scale = 0.01
	Gradient boosting	nTrees = 100, ID = 1, shrinkage = 0.1, NT = 10

C, C-parameter; ID, Interaction depth; mtry, number of features used at each split; NA, Not applicable; nTrees, Number of trees; NT, number of training set samples in a node to commence splitting. For all gradient boosting models, shrinkage and NT were held constant at 0.1 and 10, respectively.

Another important comparison included settings in which reduced sets of features were used versus settings in which no reduced sets were used. Overall, the findings suggest that using a reduced set of features seemed to somewhat improve the performance in the training set. Yet, when validating the models on the hold-out sample it seems that using a reduced set of features does not substantially contribute the performance of the models. This indicates that using a reduced set of features does not decrease performance of the models to a relevant degree, suggesting that a reduced set of features might have a similar predictive ability compared with the full set of baseline features. The confusion matrices of the best performing models in the hold-out sample within each setting can be found in Table 6.

**Table 6:** Confusion matrices of the best performing models in the hold-out sample within each setting.

		Reference	
Setting 1: Logistic regression		Non-improvement	Improvement
Predicted	Non-improvement	66	20
	Improvement	37	23
Setting 2: Gradient boosting			
Predicted	Non-improvement	69	2
	Improvement	34	22
Setting 3: Gradient boosting			
Predicted	Non-improvement	79	19
	Improvement	24	24
Setting 4: Gradient boosting			
Predicted	Non-improvement	79	21
	Improvement	24	22

### 3.5. Feature importance

To allow for some interpretation of the models, one last step was to identify the most important features from the models that showed the best performance on the hold-out sample in each setting. An overview of these five most important features can be found in Table 7. It is noteworthy that change scores seem to play a crucial role in the models that include change scores. This, again, suggests that including information about change within the beginning of treatment seems to be valuable when aiming to improve model accuracy. Furthermore, in all settings, except the second setting, both psychopathology and well-being are among the most important features. This indicates that not only psychopathology seems to be of importance when predicting improvement in symptoms, but also well-being.

**Table 7:** Five most important features of the best performing models in each setting.

	Setting 1: Logistic regression	Setting 2: Gradient boosting	Setting 3: Gradient boosting	Setting 4: Gradient boosting
Feature 1	OQ-45 symptomatic distress	OQ-45 symptomatic distress	Change score OQ-45 symptomatic distress	Change score OQ-45 symptomatic distress
Feature 2	Treatment intensity	Treatment intensity	Change score OQ-45 somatic complaints	Change score OQ-45 somatic complaints
Feature 3	OQ-45 social role performance	Number of previous treatments: 5–10	OQ-45 symptomatic distress	Change score MHC-SF total score
Feature 4	GAF score	Working problems	Change OQ-45 interpersonal relations	OQ-45 symptomatic distress
Feature 5	MHC-SF total score	Main diagnosis: anxiety	Change score MHC-SF total score	Living problems

## 4. Discussion

### 4.1. Main findings

The goal of the current study was to evaluate and compare the performance of different machine learning (ML) models in predicting non-improvement in an observational sample of patients treated in routine specialized mental healthcare. Below, the results are critically discussed in the light of previous research and opportunities for future research.

First, the ML models applied in the current study showed only modest performance in predicting treatment outcomes. Although some previous prediction studies show relatively good predictive results [e.g., (98–100)], most previous studies also indicate modest performance [e.g., (30, 53, 57, 70, 73, 101, 102)]. Some explanations for the modest performance in the current study should be considered. Firstly, ‘confounding by indication’ could have introduced a bias into the observed association of observed features and non-improvement (103). The decision to assign (intensity of) treatment or adjustments along the way can be influenced by various factors, such as disease severity, previous treatments, or patient preferences. It is possible that the predictors that drive treatment assignment, in this case confounding features, could have effected the treatment outcome and have made it difficult to assess the true predictive nature of the features considered in this study (103). Secondly, in real-world scenarios, external factors or sources of noise could have affected the outcome and introduced unpredictability. These factors may not be captured by the available features. Accounting for such factors or acquiring additional relevant data might help improve performance. Feature selection, domain expertise, or acquiring additional relevant features can potentially enhance the model’s performance. The challenge remains to add the right features predicting treatment success (104). Thirdly, in the current study treatment success is assessed based on subjective self-reported measures. The patient’s responses to outcome measures might be influenced by their desire to align their responses with the clinician’s expectations. This can result in inflated self-reported outcomes, leading to reduced accuracy in predicting treatment success. People respond inconsistently over time, but algorithms assume no response bias (105). These potential errors undermine prediction. ML techniques *per se* aren’t a panacea for higher accuracy without a quality dataset of informative and relative features and domain-specific considerations (106, 107).

Second, more complicated and flexible ML models did not perform substantially better than logistic regression. This is in line with a review of 71 clinical prediction modeling studies (108) and with a recent prediction study of eating disorder treatment response by Espel-Huynh et al. (98). One explanation for this finding might be that the feature set in the current study was not large enough for the more complex models to have an advantage over logistic regression. ML algorithms lead to better performance including in the prevention of the risk of overfit with a greater number of predictors than traditional statistical methods (109). More studies have to be conducted to investigate which model works best in which circumstances (60, 108, 110). Further research into the possibilities of ML methods is still warranted since traditional regression-related approaches have various potential limitations, such as the assumption of straightforward linearity, which may render them less suitable for investigating the complex relational patterns between varied predictors for treatment success in mental healthcare (58, 111).

Third, although still modest, models that included change scores showed the highest overall performance in the hold-out sample, with the gradient boosting model achieving the best overall performance. Models without change scores performed poorly overall. These findings suggest that including change scores substantially improves prediction performance in this setting. Improvement in the first months has often been found to be related to later treatment success in other studies as well (93, 94) and early change predicts outcome even better than patient characteristics (92, 112, 113). This underscores the relevance of continuous treatment effect monitoring and treatment adjustments in clinical practice.

Fourth, the feature-reduced models demonstrated no relevant decrease in performance for predicting treatment outcomes at 6 months in the hold-out sample. Feature-reduced models potentially prevent overfitting and increase generalizability. A trade-off exists between interpretability and accuracy when choosing algorithms. Reducing features also improves the explainability of ML based prediction models. Additional, if a reduced set of features performs equally well (or even better) in predicting non-improvement, it would also increase the practical value and implementability of such a model in daily clinical practice.

Finally, analysis of the feature importance across the different model settings suggested that the most relevant features were the 0–3 month change scores in symptomatic distress, somatic complaints, and well-being, as well as baseline symptomatic distress. The importance of monitoring both the level of psychopathology and well-being in patients with mental health problems has been demonstrated more often (81, 114–118). Crucial predictors found in prior research, including chronicity, comorbidity, interpersonal functioning and familial problems (119), seemed less relevant for predicting non-response in the current study.

For practice, past and present findings underline the importance of searching for additional features to better predict treatment effect in real-world treatment context. Hilbert et al. (73) argued previously that prediction models developed within a diagnostically homogeneous sample are not necessarily superior to a more diverse sample that includes different diagnostic groups. The current study shows that the specific main diagnosis has less predictive value than, for example, early change in treatment effect. After all, where psychiatric patients differ enormously in severity, duration or symptoms of psychopathology and in risk of recurrence, treatments in daily care differ in used methods, assumed mechanisms and appointment frequency. Even within a specific diagnostic group, tailoring psychotherapeutic interventions specifically to the circumstances and characteristics of the patient can improve treatment outcomes (16, 120, 121). Depending on the context and goal of a ML model, one might want to adjust the probability cut-off for predicting non-improvement. We decided to use a probability cut-off of 50% for predicting non-improvement, because we did assume the cost of misspecification to be equal for the positive and negative class. For example, if one wants to aim for a model that has higher sensitivity, lowering the threshold could be desirable.

### 4.2. Strengths and limitations

The current study is one of the first to explore the potential of different machine learning models to predict treatment outcomes in a real-world mental healthcare context using a wide range of routinely available sociodemographic, clinical and patient-reported outcome data. There are however some limitations to the current study that need to be considered.

First, although the current study used a cross-validation approach by randomly splitting the dataset into a training and a test sample, which is the common approach in ML, it should be noted that the study is still exploratory in nature, Although common practice in ML, the test set consisted of a random subset from the same overall patient sample and therefore the study was still limited in its ability to test the generalizability of the final models. Confirmatory studies in independent datasets from different contexts are still necessary to further examine the robustness of the prediction models (122).

Second, in the context of the routine collection of patient-reported outcome data, data is often missing during the course of the treatment process because patients have already improved sufficiently or, on the contrary, have not improved. This missing data is not at random, resulting in the ML algorithms to ultimately relate to a select and biased subpopulation that continues to receive treatment for at least a certain period of time.

Third, the features available in this study consisted largely of self-report data. For the future it would be interesting to incorporate more objective features such as psychological measurements into ML models (123, 124). Future ML studies could improve mental health predictions by adding a unique source of high-frequent and continuous data collecting using multi-modal assessment tools during the period of treatment. mHealth (mobile health) provides individuals real-time biofeedback via sensor apps in everyday devices such as smartphones or wearables on physiological or self-reported behavioral and state parameters, such as heart rate, sleep patterns, physical activity or stress levels (124–126). The combination of ML and mHealth, despite challenges in dimensionality, ethics, privacy and security, shows promise as a clinical tool for monitoring populations at risk and forms the basis for the next generation of mHealth interventions (124, 125).

Finally, though the chosen criterion of 0.5 SD for non-improvement is often used [e.g., (127–131)], a disadvantage is that this cutoff is sample-dependent. Also, an improvement of 0.5 SD does not necessarily mean that a patient has recovered in such a way that (s)he no longer has clinically relevant complaints. Future research could consider to use the Jacobson-Truax concept of the Reliable Change Index (RCI), which considers the reliability of the improvement in the context of the overall distribution that the patient is likely to belong to post-treatment (132). Patients moving reliably into the functional distribution are *recovered*. Patients are considered to have *improved* if they have made a reliable change but remain in the dysfunctional population, *unchanged* if they have not made a reliable change, and *deteriorated* if they have reliably worsened (132).

### 4.3. Clinical implications and recommendations for future research

Some recommendations can be made for future research. On the one hand, the use of sophisticated psychological data with relevant features according to the latest theoretical models may increase predictions and thereby improve decision-making on therapy indication. This could include the therapeutic relationship as a known predictor of interest (133) diagnosis specific questionnaires in addition to generics, which could mean that the case for a transdiagnostic approach may not yet have been settled, or program-specific questionnaires, appropriate to the therapy offered. On the other hand, the development of more advanced tools is necessary to detect predictors for treatment response based on high-dimensional patient data (134). Based on current research, practitioners might decide to stop or adjust a treatment. In the future, it is desirable that patients can be indicated in a more targeted manner. After all, at present ML approaches cannot yet contribute to specific individualized clinical judgments (135). We would encourage future studies to develop predictors over rather broad diagnostic patient groups and not exclude features in advance, but use the full potential of information available in patient EHRs (136). Interestingly, ML techniques offer the opportunity to study patients who are underrepresented in RCTs.

Additionally, ML has the potential to benefit mental healthcare as it can account for the interaction between many features (137). The ML techniques are suitable to detect features with the strongest predictive influence in different contexts and mutual interactions, thereby providing a combined measure of both individual and multivariate impact of each feature (138). Subsequently, based on findings, the number of features to be implemented in daily care can be substantially reduced.

To reduce response bias, improve the predictive performance of the model, and provide a more comprehensive picture of treatment success, it may be helpful to consider multiple perspectives and assessment sources. In addition, it is important to recognize and address the potential discrepancies between the assessments of different stakeholders (e.g., clinician and patient) when defining the criterion for treatment success in predictive studies.

As change scores in both psychopathology and well-being proved relevant, implementing change measurements in ML applications could be more standardized. Therefore, for future studies, we recommend that in addition to predicting changes in psychopathology, algorithms to predict non-improvements in well-being and other domain/construction should be included. Also, adding multiple change scores, such as living conditions in daily activities and social relationships, or compliance with homework-related adherence could be relevant (139). Adding other data modalities, such as the relationship with the patient’s life story, or test data could also improve prediction performance (140, 141). In any case, it is advisable to closely monitor changes in psychopathology and well-being in clinical practice and decision making from the very beginning, so that timely adjustments can be made in the therapy of non-responders. Tiemens et al. (142) recommend doing this at least 4 weeks after starting treatment. The measurement of change scores is also important because the use of feedback based on these evaluations in itself has a positive effect on complaint reduction and it can shorten the duration of treatment (143, 144).

Finally, applying both ML and traditional statistical approaches in the same study allows for comparisons (109, 145). By learning from unique strengths and limitations of different ML algorithms, future ML research can contribute to increasingly accurate predictions (146).

## 5. Conclusion

In the current study we applied ML techniques in a real-world mental healthcare patient population to predict non-improvement using sociodemographic, psychological, diagnostic and clinical data. The overall conclusion is that working with a reduced set of data, and implementing early change scores and relatively simple models gives the best results, both in terms of accuracy and broader in interpretability and applicability. Our results show that ML can be used as a step to indicate treatment change in an early stage of treatment, where it seems to be important to use psychopathology and well-being as important features. The results are encouraging and provide an important step to use patient specific and routine collected patient-level outcome data in clinical practice to help individual patients and clinicians select the right treatments. ML may help to bridge the gap between science and practice. None of the ML applications were developed to replace the clinician, but instead were designed to advance the clinicians’ skills and treatment outcome (147). ML might become part of evidence-based practice, as a source of valuable information in addition to clinical knowledge and existing research evidence.

## Data availability statement

The datasets presented in this study can be found in online repositories. The names of the repository/repositories and accession number(s) can be found below: Data, syntax and output files can be found on the Open Science Framework website (https://osf.io/xwme4/).

## Ethics statement

Ethical approval was not required for the studies involving humans because as data were collected in the context of regular care and only anonymized data were analyzed, the study did not require medical ethical approval according to Dutch law. The studies were conducted in accordance with the local legislation and institutional requirements. The participants provided their written informed consent to participate in this study.

## Author contributions

KF collected the data, organized the database, and wrote the first draft of the manuscript. KF, JK, and PK contributed substantial to the conception, design and manuscript draft, and ensuring that the work was appropriately investigated and resolved. JK implemented machine learning algorithms and statistical analysis and wrote the first method section of the manuscript. EB and GW supervised and critically reviewed the manuscript. All authors contributed to the article and approved the submitted version.
